# Impact of lateral meniscus injury on the efficacy of medial unicompartmental knee arthroplasty in patients with anteromedial osteoarthritis: a systematic review and meta-analysis

**DOI:** 10.3389/fsurg.2026.1913901

**Published:** 2026-09-22

**Authors:** Jie Zhou, Hongmin Deng, Mingjie Fu, Jinhua Guo, Qihao Jiang, Changyu Huang, Zhibing Gong

**Affiliations:** 1 Quanzhou Orthopedic Clinical Medical College, Fujian University of Traditional Chinese Medicine, Quanzhou, Fujian, China; 2 Orthopaedics, Xinyu Hospital of Traditional Chinese Medicine, Xinyu, Jiangxi, China

**Keywords:** lateral meniscus injury, medial knee osteoarthritis, meta-analysis, systematic review, unicompartmental knee arthroplasty (UKA)

## Abstract

**Objective:**

To evaluate the effect of lateral meniscus injury on the clinical outcome of medial unicompartmental knee arthroplasty (UKA) for anteromedial osteoarthritis (AMOA), and to provide evidence-based support for clinical decision-making.

**Methods:**

A systematic literature search was conducted across PubMed, Embase, Cochrane Library, Web of Science, Chinese Biomedical Literature Database (CBM), China National Knowledge Infrastructure (CNKI), Wanfang, and VIP databases from inception to March 1, 2026. Data synthesis and meta-analysis were performed using RevMan 5.4 software. Sensitivity and subgroup analyses were conducted where appropriate, and the certainty of evidence was assessed using the Grading of Recommendations Assessment, Development and Evaluation (GRADE) approach.

**Results:**

A total of 8 retrospective cohort studies comprising 1,709 patients were included. No significant differences were observed between patients with and without lateral meniscal abnormalities in postoperative knee function scores [standardized mean difference (SMD) = −0.06, 95% confidence interval (CI) (−0.26, 0.14), *P* = 0.56], postoperative knee range of motion [mean difference (MD) = 0.17°, 95% CI (−1.58°, 1.92°), *P* = 0.85], or postoperative lateral knee pain [risk ratio (RR) = 1.10, 95% CI (0.32, 3.81), *P* = 0.89]. Sensitivity analyses showed that the pooled results remained stable. No consistent increase in revision or implant failure or lateral-compartment osteoarthritis progression was identified, although evidence for these outcomes was limited by the small number of events.

**Conclusion:**

Available evidence suggests that asymptomatic or mild degenerative lateral meniscal abnormalities may not significantly impair short- to mid-term clinical outcomes after medial UKA in appropriately selected patients with AMOA. Therefore, an asymptomatic lateral meniscal abnormality alone should not necessarily be considered an absolute contraindication to medial UKA. However, these findings should not be extrapolated to symptomatic, unstable, traumatic, or root tears or to patients with advanced lateral-compartment cartilage loss.

## 1 Introduction

Knee osteoarthritis (KOA) is one of the most common degenerative disorders of the knee and predominantly affects the medial weight-bearing compartment. Anteromedial osteoarthritis (AMOA) accounts for approximately 70%–80% of cases (1). Unicompartmental knee arthroplasty (UKA) has become a preferred treatment option for patients with disease confined to a single compartment and relatively preserved lateral and patellofemoral compartments. This is mainly due to its unique advantages, including minimal surgical trauma, preservation of the original joint structure, and restoration of near normal postoperative kinematics (2). However, in clinical practice, about 30%–50% of AMOA patients have concomitant lateral meniscus injury (LMI) (3). Whether these abnormalities adversely affect the outcomes of medial UKA remains controversial. The traditional biomechanical view is that structural destruction of the lateral meniscus will change the biomechanical balance of the lower extremity, leading to a sharp increase in contact stress and accelerating the degeneration of the lateral compartment. This phenomenon may lead to premature implant failure after UKA surgery (4). However, in recent years, an increasing number of clinical cohort studies have suggested that the degenerative changes of the lateral meniscus found on preoperative magnetic resonance imaging (MRI) alone, if not accompanied by definite clinical symptoms such as lateral pain or locking, do not seem to affect the clinical outcomes following UKA (3).

Nevertheless, the available evidence remains fragmented and heterogeneous. Previous studies have evaluated different forms of lateral meniscal abnormalities, including meniscal tears, degenerative changes, intrameniscal signal abnormalities, meniscal extrusion, and histological calcification, with substantial variation in implant design, follow-up duration, and outcome assessment (3, 5–11). Consequently, it remains uncertain whether lateral meniscal abnormalities are consistently associated with postoperative functional outcomes, knee range of motion, revision or implant failure, and lateral-compartment symptoms following medial UKA. Therefore, the present systematic review and meta-analysis aimed to synthesize the available comparative evidence and evaluate the association between preoperative lateral meniscal abnormalities and postoperative outcomes after medial UKA in patients with AMOA. We hypothesized that, in appropriately selected patients with AMOA, particularly those without clinically significant symptoms attributable to the lateral meniscus, the presence of preoperative lateral meniscal abnormalities would not be associated with poorer postoperative functional outcomes, reduced knee range of motion, or an increased risk of revision, implant failure, or lateral-compartment symptoms.

## 2 Materials and methods

### 2.1 Protocol registration and reporting

This systematic review and meta-analysis was registered in the International Prospective Register of Systematic Reviews (PROSPERO; registration number: **CRD420261468960**) under the title “*Impact of Lateral Meniscus Injury on the Efficacy of Medial Unicompartmental Knee Arthroplasty in Patients with Anteromedial Osteoarthritis: A Systematic Review and Meta-Analysis.”* The review was conducted and reported in accordance with the Preferred Reporting Items for Systematic Reviews and Meta-Analyses (PRISMA) 2020 statement.

### 2.2 Eligibility criteria

The inclusion criteria were as follows: (1) **Participants:** patients with anteromedial knee osteoarthritis treated with UKA, and clinical data on meniscal injuries should be clearly reported; (2) **Study design:** retrospective cohort studies; (3) **Data requirements:** well-defined outcome measures.

The exclusion criteria included: (1) **Preclinical studies**, such as laboratory or animal experiments; (2) **Studies with insufficient data**, such as no clear outcome measures or small sample size.

### 2.3 Search strategy

The search was conducted in accordance with PRISMA 2020. We systematically searched PubMed, Embase, the Cochrane Library, Web of Science, Chinese Biomedical Literature Database (CBM), China National Knowledge Infrastructure (CNKI), Wanfang Data, and VIP Database from inception to March 1, 2026. The search strategy was based on the following keywords (“lateral meniscus injury” OR “lateral meniscus tear” OR “lateral meniscus degeneration”) AND (“unicompartmental knee arthroplasty” OR “UKA” OR “partial knee replacement”) AND (“anteromedial osteoarthritis” OR “AMOA” OR “knee osteoarthritis”). In addition, we manually searched the reference lists of the included articles to identify any further potentially eligible studies. The specific retrieval strategy will be provided as Supplementary Data Sheet S1.

### 2.4 Study selection and data extraction

Two researchers who had been trained in evidence-based methods independently screened the literature and extracted the data according to the inclusion and exclusion criteria, and then cross-checked the data. Disagreements were resolved through discussion or, when necessary, adjudication by a third investigator. A standardized data extraction form was used to collect study characteristics, including the first author, publication year, country, sample size, study design, and reported outcome measures.

### 2.5 Quality assessment of included studies

The methodological quality of the included retrospective cohort studies was assessed using the Newcastle-Ottawa Scale (NOS). Two independent investigators scored each study on three dimensions: selection of study population (0–4 points), comparability between groups (0–2 points), and ascertainment of outcomes (0–3 points). Studies with NOS scores of 7–9 were classified as high quality, those with scores of 5–6 as moderate quality, and those with scores <5 as low quality. Disagreements between the two assessors were resolved by consulting a third investigator.

Publication bias was not formally assessed using funnel plots or statistical tests for funnel plot asymmetry because fewer than 10 studies were available for each pooled outcome, rendering such assessments unreliable. Sensitivity analyses were performed by excluding studies of moderate methodological quality (NOS score = 6), where applicable, to evaluate the robustness of the pooled estimates.

### 2.6 Statistical analysis

RevMan 5.4 software was used to perform a meta-analysis of the included studies. Continuous outcomes were summarized using mean differences (MDs) or standardized mean differences (SMDs), whereas dichotomous outcomes were summarized using risk ratios (RRs) or odds ratios (ORs), with corresponding 95% confidence intervals (CIs). All results were reported with their corresponding 95% confidence intervals (CIs). Statistical heterogeneity was assessed using the I² statistic and Cochran's Q test. When *P* > 0.10 and I² < 50%, heterogeneity was considered low and a fixed-effect model was used; otherwise, a random-effects model was applied.

Post hoc exploratory subgroup analyses were performed according to follow-up duration (<3 years vs. ≥3 years) to investigate whether the duration of postoperative observation influenced the pooled functional and range-of-motion outcomes. Differences between subgroups were evaluated using the test for subgroup differences. Implant-bearing design (mobile-bearing vs. fixed-bearing) was also considered as a potential source of clinical heterogeneity. However, formal bearing-specific subgroup meta-analysis was performed only when sufficient independent studies were available within each subgroup. When one bearing category was represented by only one study or when all studies contributing to an outcome used the same implant design, the findings were summarized descriptively because a formal subgroup comparison would be statistically unstable and potentially misleading.

For rare outcomes with very few events and double-zero studies, quantitative pooling was considered statistically unstable; these outcomes were therefore summarized descriptively.

### 2.7 Certainty of evidence

The certainty of evidence for each principal outcome was assessed using the Grading of Recommendations Assessment, Development and Evaluation (GRADE) approach. Because preoperative lateral meniscal abnormality was considered an exposure/prognostic factor rather than an assigned intervention, the assessment followed GRADE guidance for prognostic-factor evidence. The body of observational evidence was initially rated as high certainty and was subsequently evaluated for potential downgrading across five domains: risk of bias, inconsistency, indirectness, imprecision, and publication bias. The certainty of evidence was categorized as high, moderate, low, or very low. Two reviewers independently performed the assessment, and disagreements were resolved through discussion or consultation with a third reviewer. Detailed judgments and reasons for downgrading are presented in Table 3.

## 3 Results

### 3.1 Literature search results

A total of 209 potentially relevant records were identified through the initial comprehensive database search, comprising 127 in English and 82 in Chinese. After the removal of 64 duplicate records, the titles and abstracts of the remaining 145 articles were screened, resulting in the exclusion of 102 studies that failed to meet the inclusion criteria. Subsequently, a full-text review of the remaining 43 articles was conducted, leading to the further exclusion of 35 articles that did not satisfy the eligibility requirements. Ultimately, 8 studies were deemed eligible and included in the final systematic review and meta-analysis, as shown in Figure 1.

**Figure 1:**
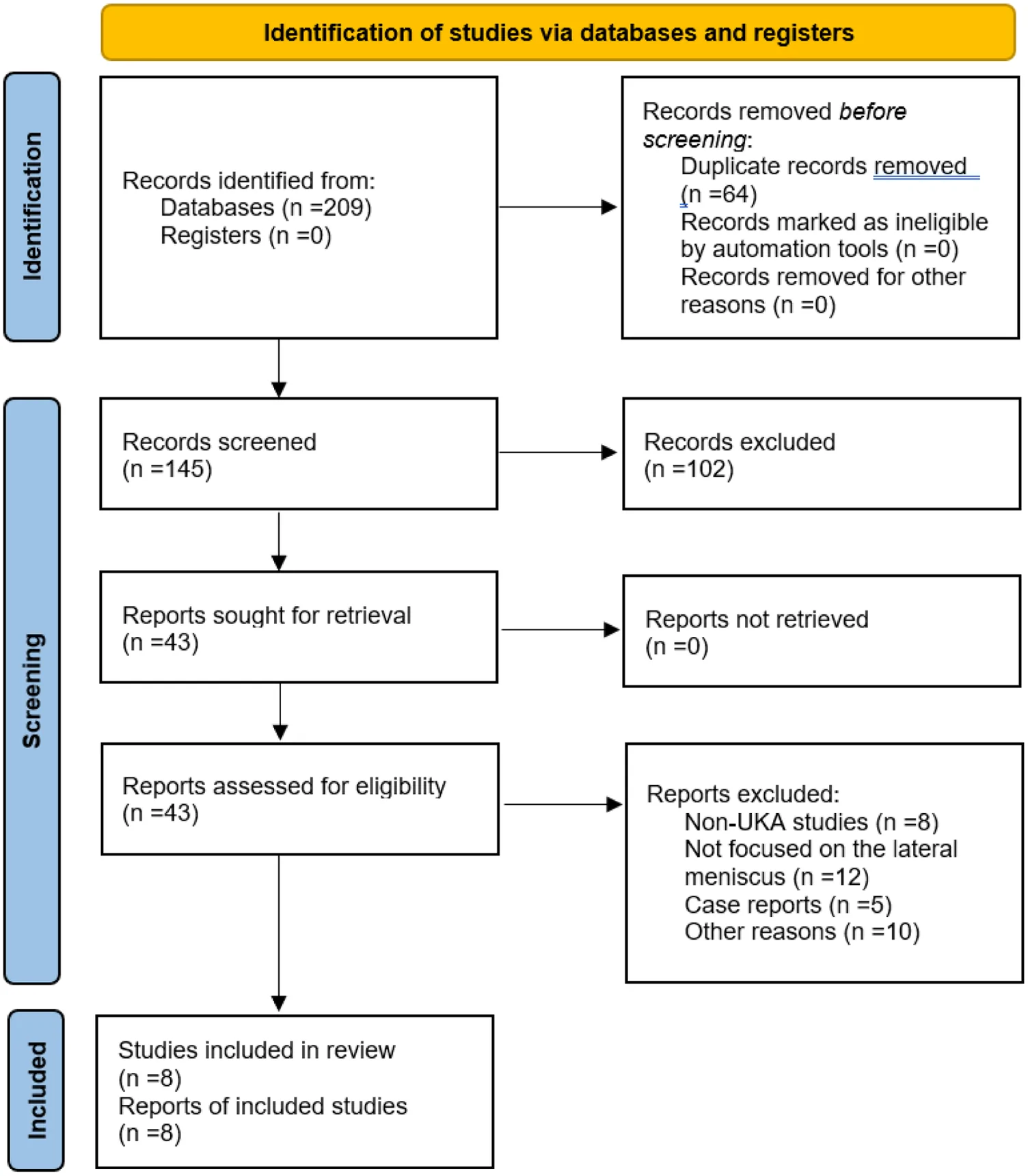
PRISMA 2020 flow diagram for study identification and selection.

### 3.2 Baseline characteristics and methodological quality assessment

A total of 8 studies (3, 5–11) were ultimately included, comprising a pooled sample size of 1,709 patients. Among them, 1,341 cases were in the intact lateral meniscus group (normal group), and 368 cases were in the lateral meniscus injury group (abnormal group). All included studies reported clearly defined outcome measures. The baseline characteristics of the included literature are summarized in Table 1. The methodological quality of the eight included retrospective cohort studies was rigorously evaluated using the Newcastle-Ottawa Scale (NOS). 6 studies (3, 5–9) were classified as high-quality (NOS score ≥ 7), while the remaining 2 studies (10, 11) were deemed moderate-quality (NOS score = 6). Point deductions were primarily attributed to the relatively short follow-up durations in certain studies with smaller sample sizes, as well as inadequate control for confounding variables.

**Table 1:** Baseline characteristics of the included studies (*n* = 8).

First Author (Year)	Country	Study design	Sample Size (n) (Intact/LMI)	Mean follow-up	UKA implant type	Outcome measures
Park (2021) (5)	South Korea	Retrospective cohort	125 (59/66)	74.4 months	Fixed bearing	KSS, KOOS, lateral pain, squatting ability, survivorship, revision rate
Hernigou et al. (2023) (6)	France/China	Retrospective cohort	130 (60/70)	10 years	Fixed bearing	KSS, lateral radiographic progression (JSN), 10-year survivorship, revision rate
Zhu et al. (2023) (3)	China	Retrospective cohort	104 (59/45)	39.2 months	Mobile bearing	HSS, ROM, lateral knee pain, squatting/extension ability, complications, revision rate
Hurst et al. (2013) (10)	USA	Retrospective cohort	1,000 (967/33)	43.4 months (LMI)/38.3 months (Intact)	Mobile bearing	KSS (pain, clinical, function), failure rate, revision rate
Fukai et al. (2025) (8)	Japan	Retrospective cohort	117 (98/19)	> 3 years	Mobile bearing	OKS, KSS-F, ROM, lateral OA progression, revision rate
Tanaka et al. (2020) (9)	Japan	Retrospective cohort	102 (39/63)	3 years	Mobile bearing	OKS, KSS-F, ROM, reoperation rate, revision rate
Chen et al. (2023) (7)	China	Retrospective cohort	101 (44/57)	∼2 years	Mobile bearing	AKSS-F, AKSS-O, OKS, ROM, HKA angle, complications, revision rate
Mao et al. (2025) (11)	China	Retrospective cohort	30 (15/15)	13.9 months	Mobile bearing	HSS, ROM, lateral knee pain, operative time, complications

KSS, Knee Society Score; KOOS, Knee Injury and Osteoarthritis Outcome Score; HSS, Hospital for Special Surgery score; OKS, Oxford Knee Score; KSS-F, Knee Society Score-Function; AKSS-F, American Knee Society Score-Function; AKSS-O, American Knee Society Score-Objective; ROM, range of motion; JSN, joint space narrowing; HKA, hip-knee-ankle; OA, osteoarthritis.

To improve consistency in the interpretation of lateral meniscal pathology, the study-specific definitions of lateral meniscal abnormalities were extracted and harmonized into standardized descriptive phenotypes, including structural meniscal injury/tear, degenerative or intrameniscal signal abnormality, meniscal extrusion, tear/calcification-related abnormality, and broader MRI abnormalities not specific to isolated LMI (3, 5–11). The original definitions reported by each study were retained to avoid implying biological equivalence among different lesion types.

Symptom status was independently classified as (1) asymptomatic/clinically silent, defined as the absence of clinically relevant lateral joint-line pain, locking, or other mechanical symptoms attributable to the lateral meniscus; (2) symptomatic, when clinically relevant lateral meniscus-related symptoms were explicitly reported; or (3) unclear/not reported when symptom status could not be reliably determined from the original study. Because symptom status was inconsistently reported and no sufficient set of studies provided an independently analyzable symptomatic LMI cohort, symptomatic-versus-asymptomatic quantitative subgroup meta-analysis was not considered appropriate. Instead, a stratified descriptive analysis was performed. The specific results are shown in Table 2.

**Table 2:** Study-specific LMI phenotype and symptom status.

Study	Original definition of LMI	Standardized phenotype	Severity information	Symptom status	Recommended use
Park (5)	Stoller 1–3; degeneration/intrasubstance, horizontal, complex, radial, discoid lesions	Mixed degenerative signal + definite tear	Mixed; grade-specific outcome unavailable	Asymptomatic	Asymptomatic subgroup
Hernigou (6)	Meniscal tear ± histological calcification	Tear/calcification	Phenotype-based, not ordinal	Unclear	Separate descriptive category
Zhu (3)	Stoller grade 3 vs. grade 0	Definite structural tear	Grade 3: *n* = 45	Unclear	Definite-tear subgroup
Hurst (10)	Abnormal preoperative MRI	Abnormal meniscus MRI	Mixed; grade-specific outcome unavailable	Unclear	Indirect evidence; separate descriptive interpretation
Fukai (8)	Lateral meniscal extrusion	Extrusion	Grade 1: 18; Grade 2: 1	Unclear	Separate extrusion category
Tanaka (9)	Stoller grades 1–3	Degeneration/definite tear	G1 = 40, G2 = 15, G3 = 8	Unclear	Severity analysis
Chen (7)	Stoller grades 1–3	Degeneration/definite tear	G1 = 38, G2 = 13, G3 = 6	Unclear	Main severity-stratified study
Mao (11)	Fischer II–III	MRI-defined structural LMI	II = 2, III=13	Asymptomatic	Asymptomatic subgroup

Across the assessed domains, concerns regarding exposure ascertainment and outcome assessment were generally low. However, methodological limitations remained with respect to study comparability and adequacy of follow-up. Several smaller cohorts did not use multivariable adjustment or propensity-score methods, resulting in incomplete control of potential confounding. In addition, some studies had relatively short follow-up periods. Overall, the methodological quality of most included studies was considered acceptable. Sensitivity analyses were subsequently performed to assess whether studies of moderate methodological quality materially influenced the pooled estimates. The results are summarized in Figures 2a,b.

**Figure 2:**
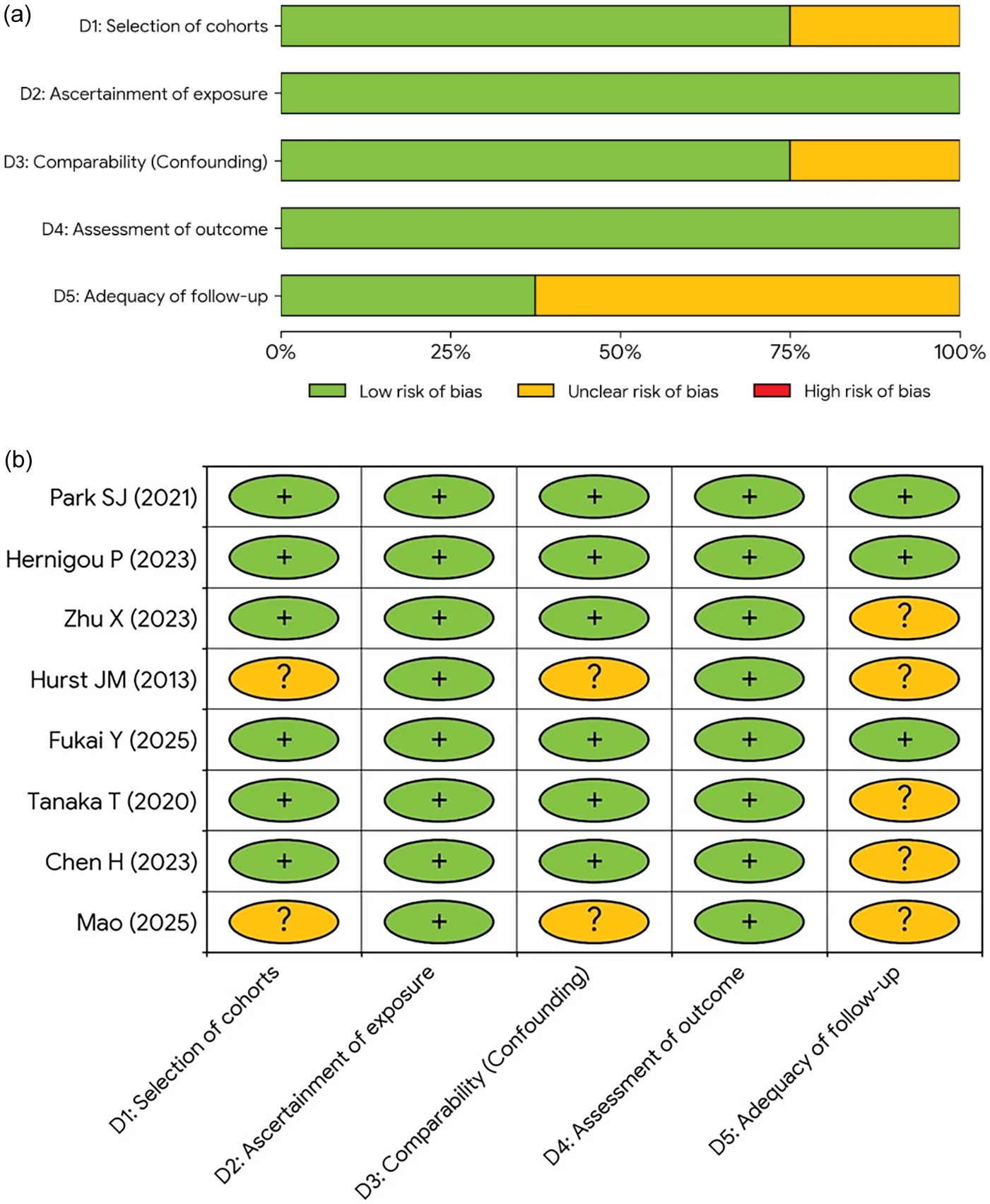
**(a)** Methodological quality and risk of bias assessment using the Newcastle-Ottawa Scale. Risk of bias graph across all domains. **(b)** Methodological quality and risk of bias assessment using the Newcastle-Ottawa Scale. Risk of bias summary for each study.

### 3.3 Primary quantitative meta-analyses

#### 3.3.1 Postoperative functional scores

Five studies (3, 7–9, 11) evaluated postoperative functional outcomes after medial UKA in patients with and without lateral meniscal abnormalities. Because different functional scoring systems were used across studies, standardized mean differences (SMDs) were calculated. No substantial statistical heterogeneity was observed (I² = 0%), and a fixed-effect model was applied. The pooled analysis showed no statistically significant difference between the LMI and intact groups (SMD = −0.06, 95% CI −0.26–0.14, *P* = 0.56; I² = 0%) (Figure 3). The magnitude of the pooled effect was small and close to the null, suggesting that any average difference in postoperative functional outcomes was likely to be limited.

**Figure 3:**
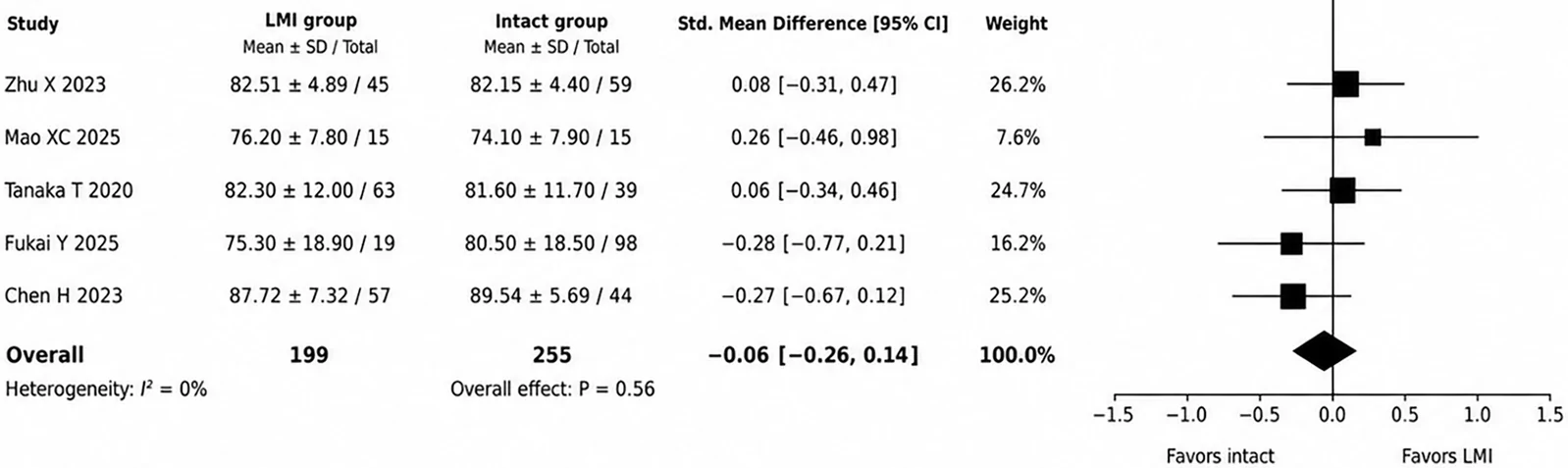
Forest plot illustrating the impact of lateral meniscus injury on postoperative functional scores after medial UKA.

#### 3.3.2 Postoperative knee range of motion (ROM)

Four studies (7–9, 11) provided comparable postoperative knee range-of-motion data. No substantial heterogeneity was detected (I² = 0%), and a fixed-effect model was used. The pooled estimate showed no statistically significant difference in postoperative ROM between patients with and without lateral meniscal abnormalities (MD = 0.17°, 95% CI −1.58°–1.92°, *P* = 0.85; I² = 0%) (Figure 4). The absolute pooled difference was small and was unlikely to represent a clinically important difference in routine practice.

**Figure 4:**
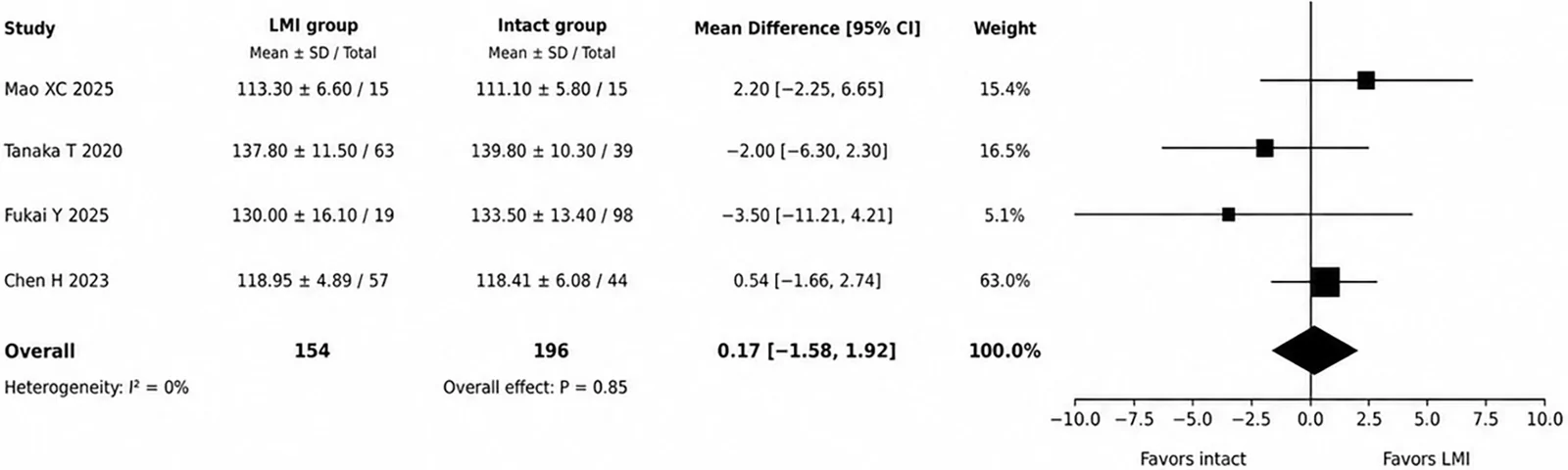
Forest plot illustrating the impact of lateral meniscus injury on postoperative knee range of motion (ROM) after medial UKA.

#### 3.3.3 Postoperative lateral knee pain

Three studies provided comparable data regarding postoperative lateral knee pain (3, 5, 11). No substantial statistical heterogeneity was detected (I² = 0%), and a fixed-effect model was used. The pooled analysis showed no statistically significant association between preoperative lateral meniscal abnormalities and postoperative lateral knee pain (RR = 1.10, 95% CI 0.32–3.81, *P* = 0.89; I² = 0%) (Figure 5). However, the confidence interval was wide and encompassed both a potentially meaningful reduction and increase in risk; therefore, the absence of statistical significance should not be interpreted as evidence of clinical equivalence.

**Figure 5:**
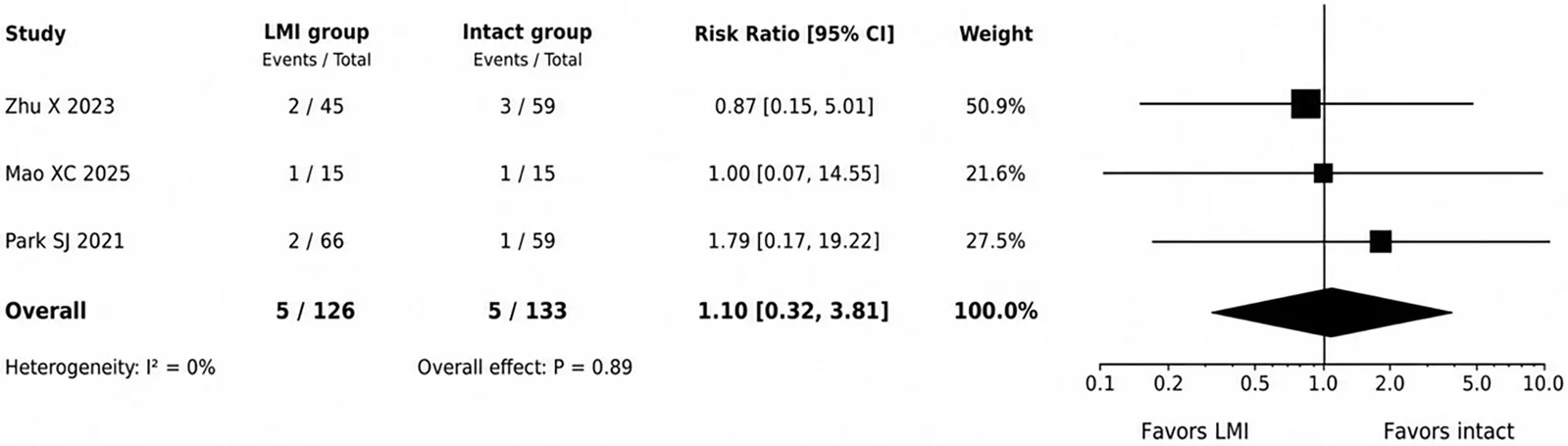
Forest plot of postoperative lateral knee pain after medial unicompartmental knee arthroplasty.

### 3.4 Sensitivity and exploratory subgroup analyses

#### 3.4.1 Sensitivity analyses

Among the two moderate-quality studies, only Mao et al. (11) contributed to the primary quantitative meta-analyses; Hurst et al. (10) did not contribute data to these pooled functional, ROM, or lateral knee pain analyses. Sensitivity analyses were performed by excluding studies rated as having moderate methodological quality (NOS score = 6), where applicable. After exclusion of Mao et al., the pooled estimate for postoperative functional scores remained non-significant (SMD = −0.09, 95% CI −0.29–0.12, *P* = 0.42; I² = 0%) (Figure 6A). Similarly, exclusion of Mao et al. did not materially alter the pooled estimate for postoperative knee ROM (MD = −0.20°, 95% CI −2.10°–1.70°, *P* = 0.84; I² = 0%) (Figure 6B) or postoperative lateral knee pain (RR = 1.12, 95% CI 0.28–4.59, *P* = 0.87; I² = 0%) (Figure 6C). Overall, exclusion of the moderate-quality study did not materially alter the direction or statistical significance of the primary quantitative findings, although the confidence interval for lateral knee pain remained wide.

**Figure 6:**
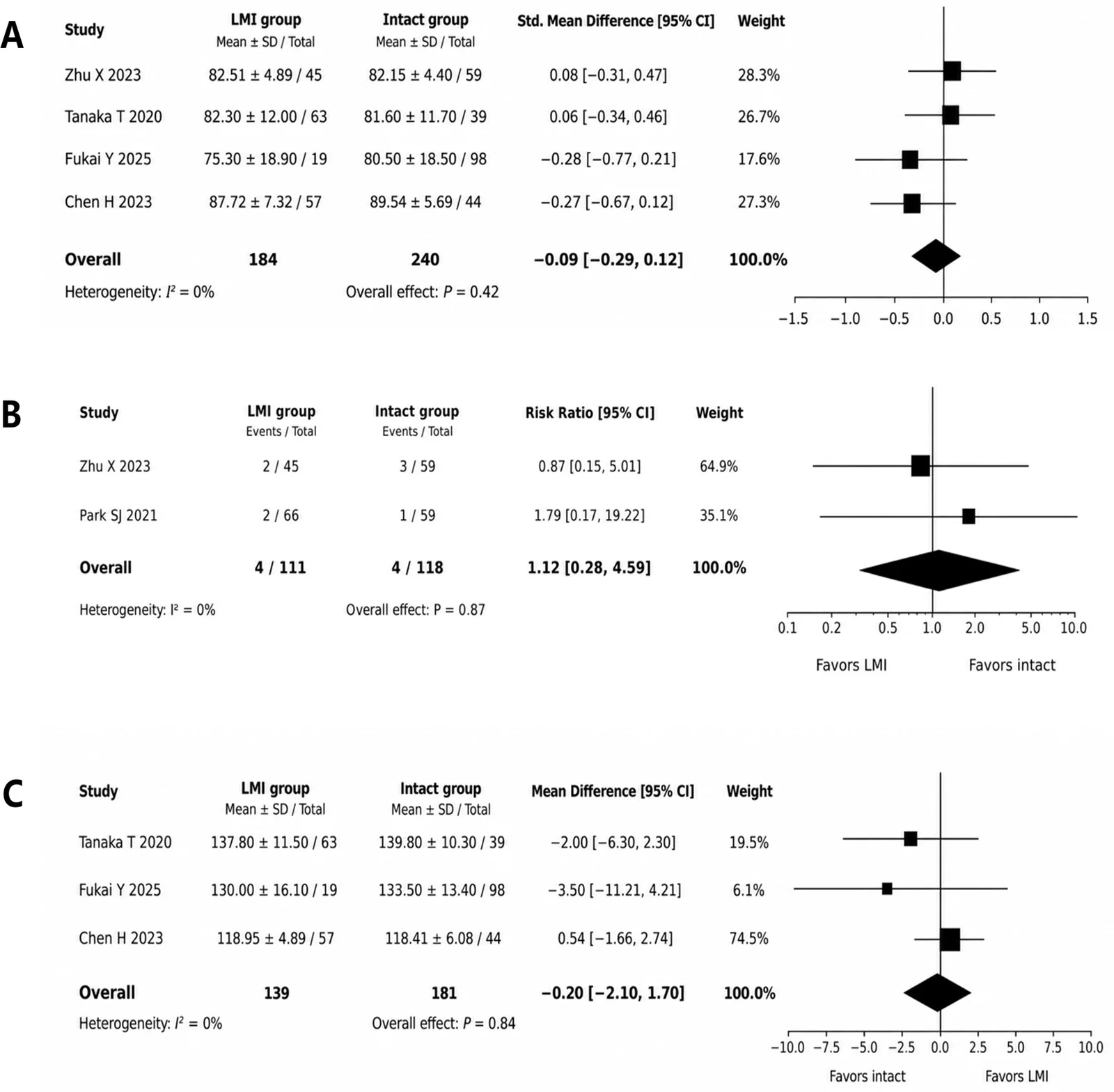
**(A)** Sensitivity analyses of postoperative functional scores after medial unicompartmental knee arthroplasty. **(B)** Sensitivity analyses of knee range of motion after medial unicompartmental knee arthroplasty. **(C)** Sensitivity analyses of lateral knee pain after medial unicompartmental knee arthroplasty.

#### 3.4.2 Exploratory subgroup analyses

Exploratory subgroup analyses were performed according to follow-up duration (<3 years vs. ≥3 years). For postoperative functional scores, the pooled estimate was SMD = −0.15 (95% CI −0.49–0.20) in studies with follow-up <3 years and SMD = −0.02 (95% CI −0.26–0.23) in studies with follow-up ≥3 years. No statistically significant subgroup difference was detected (*P* for subgroup difference = 0.54) (Figure 7A).

**Figure 7:**
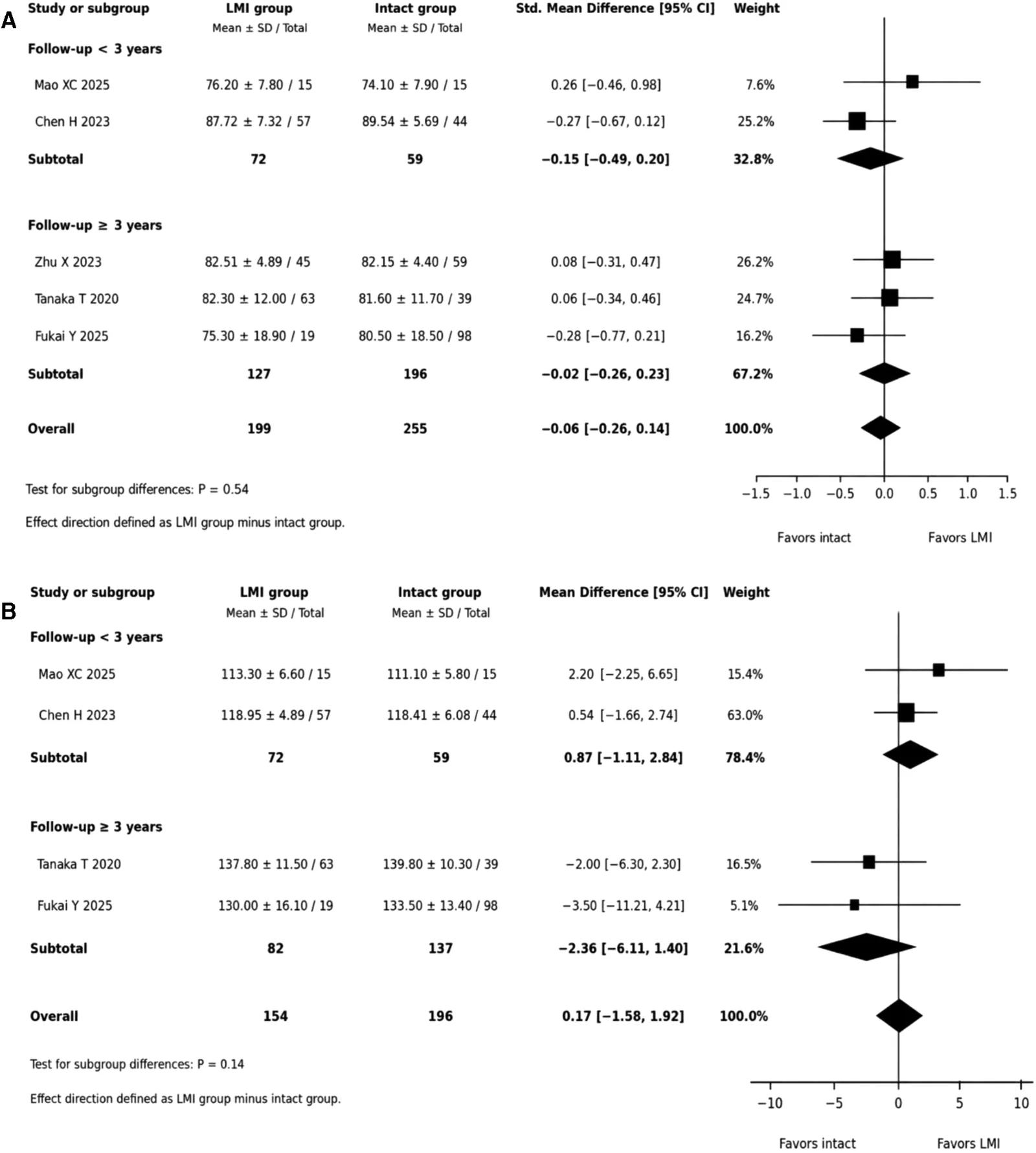
**(A)** Functional score subgroup by follow-up. **(B)** ROM subgroup by follow-up.

For postoperative knee ROM, the pooled estimate was MD = 0.87° (95% CI −1.11°–2.84°) in studies with follow-up <3 years and MD = −2.36° (95% CI −6.11°–1.40°) in studies with follow-up ≥3 years. The difference between the two follow-up subgroups was also not statistically significant (*P* for subgroup difference = 0.14) (Figure 7B).

These exploratory analyses did not demonstrate clear evidence that follow-up duration modified the pooled functional or ROM effects. However, the subgroup findings should be interpreted cautiously because of the small number of studies within each subgroup and the *post hoc* nature of the 3-year cutoff.

Subgroup analysis according to implant-bearing design was also considered. However, all studies contributing to the pooled functional-score and range-of-motion analyses used mobile-bearing UKA (3, 7–9, 11), precluding a direct comparison with fixed-bearing implants for these outcomes. For postoperative lateral knee pain, fixed-bearing UKA was represented by only one study in the quantitative analysis, making a formal between-bearing subgroup comparison statistically unreliable. Revision or implant failure was summarized descriptively because of the small number of events and the presence of double-zero studies; therefore, a bearing-specific quantitative subgroup analysis was not performed. Accordingly, differences in implant design were summarized descriptively rather than quantitatively.

Similarly, geographic subgroup analysis was not undertaken because the small number of studies and the limited number of studies within individual geographic regions precluded statistically meaningful between-region comparisons.

#### 3.4.3 Stratified descriptive analysis according to LMI phenotype and symptom status

The definitions of lateral meniscal abnormalities varied substantially across the included studies and were therefore harmonized into descriptive phenotypic categories while retaining the original study-specific definitions. These included structural meniscal injury or tear, degenerative or intrameniscal signal abnormality, meniscal extrusion, tear/calcification-related abnormalities, and broader abnormal MRI findings. Hurst et al. was categorized separately as providing indirect evidence because the abnormal MRI group was not restricted to isolated lateral meniscal pathology.

With respect to symptom status, Park and Mao et al. provided evidence that could be classified as predominantly asymptomatic or clinically silent lateral meniscal abnormalities. In these studies, lateral meniscal abnormalities were not accompanied by clinically significant lateral meniscus-related pain or locking, and no clear adverse association with postoperative outcomes was identified (5, 11).

No included study provided a distinct and independently analyzable cohort consisting of patients with clearly symptomatic lateral meniscal pathology, such as persistent lateral joint-line pain or mechanical locking attributable to an unstable or root tear. The remaining studies either did not report symptom status sufficiently or primarily classified patients according to imaging or histological morphology. Consequently, a quantitative comparison between symptomatic and asymptomatic LMI could not be performed. The findings from studies with unclear symptom status were therefore interpreted separately and were not considered direct evidence supporting medial UKA in patients with symptomatic lateral meniscal lesions.

### 3.5 Secondary outcomes: descriptive synthesis

#### 3.5.1 Revision or implant failure

Revision or implant failure events were uncommon across the included studies (3, 5–10). Several studies reported no revision events in either comparison group, whereas only a small number of events were observed in the remaining studies. The available evidence was further limited by substantial imbalance in group size and differences in the definition of lateral meniscal abnormalities, including the broader abnormal-MRI classification used by Hurst et al. (10). Given the very small number of events, double-zero studies, and resulting statistical imprecision, revision or implant failure was summarized primarily as a secondary descriptive outcome rather than used as a principal quantitative endpoint. Overall, no consistent signal of an increased revision or implant-failure risk associated with lateral meniscal abnormalities was identified; however, clinically important differences cannot be excluded.

#### 3.5.2 Radiographic progression of lateral-compartment osteoarthritis

Radiographic progression of lateral-compartment osteoarthritis was inconsistently reported and was therefore summarized descriptively. Fukai et al. and Tanaka et al. reported no lateral-compartment OA progression in either comparison group during short- to mid-term follow-up (8, 9). Hernigou et al. reported a small number of cases of lateral-compartment joint-space narrowing during longer-term follow-up; however, the classification of lateral meniscal pathology in that study differed from the binary LMI-versus-intact definitions used in other cohorts (6). Because outcome definitions, exposure classifications, and follow-up durations were not sufficiently comparable, no pooled effect estimate was considered clinically appropriate. The available evidence is therefore insufficient to establish whether preoperative lateral meniscal abnormalities influence long-term radiographic progression of the lateral compartment.

### 3.6 Publication bias

Formal assessment of publication bias using funnel plots or statistical tests for funnel plot asymmetry was not performed because fewer than 10 studies contributed to each meta-analysis. Given the small number of studies per outcome, such analyses would have had insufficient power to reliably distinguish publication bias from chance-related asymmetry.

### 3.7 Certainty of evidence

The certainty of evidence was assessed using the GRADE approach. Evidence for postoperative functional scores and knee range of motion was rated as low certainty, primarily because of concerns regarding risk of bias and indirectness arising from heterogeneous definitions of lateral meniscal abnormalities. Evidence regarding postoperative lateral knee pain was rated as very low certainty because of additional imprecision associated with the small number of events and wide confidence intervals. Evidence for revision or implant failure was also rated as very low certainty because of risk of bias, indirectness, and very serious imprecision. In particular, Hurst et al. used a broader definition of abnormal preoperative MRI findings rather than isolated lateral meniscal injury, reducing the directness of the evidence for this outcome. Evidence regarding radiographic progression of lateral-compartment osteoarthritis was likewise rated as very low certainty because of the limited number of informative events and available studies. Detailed GRADE judgments are presented in Table 3.

**Table 3:** GRADE assessment of the certainty of evidence for the main outcomes.

Outcome	Studies/Participants	Effect estimate	Risk of bias	Inconsistency	Indirectness	Imprecision	Publication bias	Certainty
Postoperative functional scores	5/454	SMD = −0.06 (95% CI −0.26–0.14)	Serious^a^	Not serious^b^	Serious^c^	Not serious^d^	Not formally assessed^e^	Low ⊕⊕◯◯
Postoperative knee ROM	4/350	MD = 0.17° (95% CI −1.58°–1.92°)	Serious^a^	Not serious^b^	Serious^c^	Not serious^d^	Not formally assessed^e^	Low ⊕⊕◯◯
Postoperative lateral knee pain	3/259	RR = 1.10 (95% CI 0.32–3.81)	Serious^a^	Not serious^b^	Serious^c^	Serious^f^	Not formally assessed^e^	Very low ⊕◯◯◯
Revision or implant failure	7/1,679	No reliable pooled estimate; descriptive synthesis	Serious^g^	Not assessable^h^	Serious^i^	Very serious^j^	Not formally assessed^e^	Very low ⊕◯◯◯
Radiographic progression of lateral-compartment OA	3/349	No reliable pooled estimate; descriptive synthesis	Serious^a^	Not assessable^h^	Serious^c^	Very serious^j^	Not formally assessed^e^	Very low ⊕◯◯◯

a Risk of bias: Downgraded by one level because all contributing studies were retrospective cohort studies, with potential selection bias, residual confounding, and inconsistent adjustment for relevant prognostic variables.

b Inconsistency: Not downgraded because statistical heterogeneity was negligible (I² = 0%) and no important inconsistency in the direction of effects was identified.

c Indirectness: Downgraded by one level because the definition of lateral meniscal abnormality varied across studies, including meniscal tears, degenerative changes, intrameniscal signal abnormalities, meniscal extrusion, and histological calcification.

d Imprecision: Not downgraded because the confidence intervals were relatively narrow and did not encompass large clinically important effects.

e Publication bias: Formal assessment using funnel plots or statistical tests for funnel-plot asymmetry was not performed because fewer than 10 studies contributed to each quantitative outcome. Publication bias therefore could not be reliably assessed, and no additional downgrading was applied solely on this basis.

f Imprecision: Downgraded by one level because the confidence interval was wide and encompassed both a potentially clinically important reduction and increase in risk.

g Risk of bias for revision or implant failure: Downgraded by one level because the evidence was derived entirely from retrospective cohort studies, with potential selection bias and residual confounding, and rare-event estimates were particularly vulnerable to instability.

h Inconsistency: Could not be reliably assessed because the outcome was synthesized descriptively rather than quantitatively and the available event data were sparse.

i Indirectness for revision or implant failure: Downgraded by one level because Hurst et al. (10) evaluated a broader category of abnormal preoperative MRI findings rather than isolated lateral meniscal injury.

j Very serious imprecision: Downgraded by two levels because of the very small number of outcome events and insufficient information to exclude clinically important increases or decreases in risk.

## 4 Discussion

### 4.1 Redefining the indications for UKA

Conventional UKA strictly follows the classical indications proposed by Kozinn and Scott in 1989 (12). The complete normal structure of the lateral compartment (including the lateral meniscus) is considered to be a prerequisite for the success of the medial UKA. However, with advancing age, patients with anteromedial osteoarthritis (AMOA) frequently present with concomitant asymptomatic degenerative lateral meniscal injuries (such as horizontal or degenerative tears) (13). Consequently, in routine clinical practice, when a lateral meniscus injury is detected via preoperative magnetic resonance imaging (MRI) or intraoperative exploration, many surgeons perceive it as a definitive contraindication for medial UKA, thereby upgrading the surgical strategy to a total knee arthroplasty (TKA).

The findings of the present study corroborate that such primary or degenerative lateral meniscus injuries should no longer be regarded as an absolute contraindication for medial UKA. The pooled estimates were not only statistically non-significant but were also small in magnitude. For postoperative functional outcomes, the pooled effect estimate was close to the null (SMD = −0.06, 95% CI −0.26–0.14), suggesting that any average difference between patients with and without lateral meniscal abnormalities is likely to be small. Similarly, the pooled difference in postoperative knee range of motion was only 0.17° (95% CI −1.58°–1.92°), a magnitude that is unlikely to represent a clinically important difference in routine practice. These findings suggest that, in appropriately selected patients, the presence of preoperative lateral meniscal abnormalities alone may have limited clinical relevance for short- to mid-term postoperative function after medial UKA. However, because different functional scoring systems were used across studies, a uniform minimal clinically important difference could not be applied to the pooled standardized effect. Similarly, Zhu et al. demonstrated that lateral meniscus injuries detected on preoperative MRI do not compromise the mid-term efficacy of UKA (3). Furthermore, Tanaka et al. reached a consistent conclusion, reporting that preoperative degenerative changes of the lateral meniscus are not associated with adverse mid-term clinical outcomes following UKA (9).

An important consideration is that the definition and severity of lateral meniscal abnormalities were not uniform across the included studies. The evaluated abnormalities encompassed a broad spectrum of findings, including MRI-detected meniscal tears, degenerative changes, intrameniscal signal abnormalities, meniscal extrusion, and histological calcification (3, 5–9, 11). In addition, Hurst et al. evaluated a broader category of abnormal preoperative MRI findings rather than isolated lateral meniscal injury (10). These abnormalities should not necessarily be regarded as a single homogeneous clinical entity, because an incidental degenerative signal change or a stable horizontal tear may have substantially different biomechanical and clinical implications from a symptomatic unstable tear, a meniscal root tear, or a meniscal lesion associated with advanced lateral-compartment cartilage damage. Furthermore, the severity, tear morphology, stability, symptom status, and associated lateral cartilage condition were not consistently reported across studies. Consequently, the pooled estimates should be interpreted as reflecting predominantly degenerative or morphological lateral meniscal abnormalities rather than all types and severities of lateral meniscal injury. The stratified descriptive analysis further demonstrated that the most directly applicable evidence was derived from patients with asymptomatic or clinically silent lateral meniscal abnormalities (5, 11). In contrast, no included study provided sufficient comparative evidence specifically addressing clinically symptomatic lateral meniscal tears. Notably, only two included studies explicitly identified the evaluated lateral meniscal abnormalities as asymptomatic; symptom status was unclear or incompletely reported in the remaining cohorts. Therefore, the present findings should not be interpreted as demonstrating the safety or equivalence of medial UKA in patients with symptomatic lateral meniscal pathology. Accordingly, these findings suggest that asymptomatic or mild degenerative lateral meniscal abnormalities identified on preoperative MRI should not necessarily be considered an absolute contraindication to medial UKA when the lateral articular cartilage is preserved and no clinically significant lateral-compartment symptoms are present. However, the available evidence does not support extending this conclusion to symptomatic, unstable, root, or traumatic lateral meniscal tears or to patients with advanced lateral-compartment cartilage loss.

### 4.2 Exploration of biomechanical mechanisms

Conventional wisdom holds that structural disruption of the meniscus leads to an imbalance in the lower limb alignment, resulting in increased joint contact stress. This altered stress distribution has been hypothesized to accelerate lateral-compartment degeneration and contribute to premature implant failure after UKA (4). However, the available evidence regarding revision or implant failure in the present review does not provide a consistent signal of increased risk. Hernigou et al. reported that lateral menisci with tears or histological calcification did not increase the risk of lateral osteoarthritis after medial UKA (6). Furthermore, investigations by Chen et al. (7) regarding intrameniscal signal abnormalities, alongside evaluations by Fukai et al. on lateral meniscus extrusion, both indicate that these morphological alterations do not exert a severely detrimental effect on the postoperative function of mobile-bearing UKA, provided they do not elicit overt mechanical symptoms such as joint locking (7, 8).

The fundamental biomechanical principle of medial UKA is slight “under-correction” (typically retaining a mild varus alignment of 2°–3°), rather than overcorrection into valgus (14). Provided that intraoperative valgus overcorrection is avoided, the mechanical load on the lateral compartment does not increase significantly (15). Consequently, even in the presence of a certain degree of lateral meniscal tearing, the absence of excessive lateral stress allows the meniscus to remain in a stable, “quiescent” (dormant) state, thereby not accelerating the degeneration of the lateral cartilage (9). Because UKA preserves normal cruciate ligaments especially particularly the anterior cruciate ligament (ACL), and maintains the relative rolling and sliding of the knee joint (Rollback mechanism), this feature allows the shear force of the lateral compartment to be maintained at a low level, mechanically protecting the injured lateral meniscus.

### 4.3 Influence of implant design and follow-up duration

Differences in UKA implant design may also have influenced the pooled results. Most studies included in the analyses of postoperative functional scores and knee range of motion used mobile-bearing UKA (3, 7–9, 11), whereas fixed-bearing implants were represented in a smaller number of studies (5, 6). Mobile- and fixed-bearing designs differ in conformity, polyethylene mobility, contact mechanics, and their potential sensitivity to component positioning and soft-tissue balance. Therefore, the mechanical interaction between a pre-existing lateral meniscal abnormality and the reconstructed knee may not be identical across implant designs. Because only a limited number of studies used fixed-bearing implants and the distribution of implant designs was highly unbalanced, a meaningful implant-specific subgroup meta-analysis could not be performed. Consequently, the present pooled estimates are driven predominantly by evidence from mobile-bearing UKA and should not automatically be assumed to apply equally to all implant designs.

Follow-up duration represents another important source of clinical heterogeneity. Follow-up ranged from approximately 13.9 months in Mao et al. to 10 years in Hernigou et al. (3, 5–11), while most studies had short- to mid-term follow-up of approximately 2–6 years. This variation is particularly relevant because functional recovery and range of motion are generally detectable relatively early after UKA, whereas lateral-compartment osteoarthritis progression, polyethylene wear, aseptic loosening, and implant revision may require considerably longer periods to become clinically apparent. Therefore, studies with shorter follow-up may adequately capture early functional outcomes but may underestimate late lateral-compartment degeneration or implant-related failure. The pooled findings should consequently be interpreted primarily as evidence regarding short- to mid-term clinical outcomes, whereas conclusions regarding long-term survivorship and lateral-compartment progression remain uncertain.

### 4.4 Update on treatment paradigms

Based on the available evidence, medial UKA may remain a reasonable treatment option for appropriately selected patients with degenerative or non-incarcerated lateral meniscal abnormalities on preoperative MRI, provided that symptoms remain predominantly anteromedial, clinically significant lateral joint-line pain or locking is absent, and lateral-compartment cartilage is relatively preserved (5). Therefore, an asymptomatic MRI-detected lateral meniscal abnormality alone should not necessarily prompt conversion from medial UKA to TKA. Park reported that in the absence of lateral meniscus-related pain or locking symptoms, medial UKA can be safely performed despite the presence of abnormal MRI signals (5). However, patients with symptomatic, unstable, traumatic, or root tears, particularly when accompanied by substantial lateral cartilage loss, require separate clinical consideration and are not adequately represented by the available evidence. If the patient has definite symptoms of lateral locking before surgery, or imaging suggests that the lateral meniscus root tear is accompanied by the loss of the full thickness of the lateral cartilage, it should be reevaluated and TKA used if necessary (17).

During the medial UKA procedure, strictly avoiding medial over-stuffing is crucial for ensuring the long-term survivorship of the lateral compartment (16). If there is a free unstable flap tear of the lateral meniscus during intraoperative exploration, limited repair may be performed through a small incision or with adjunctive arthroscopy. Stable degenerative tears may be left untreated to avoid unnecessary meniscectomy and preserve residual meniscal function (3).

### 4.5 Limitations

This study has several limitations. First, all included studies were retrospective cohort studies, which inherently limits causal inference and introduces potential selection bias and confounding. Patient selection for medial UKA was based on routine clinical practice rather than random allocation, and surgeons may have preferentially selected patients with milder, stable, or asymptomatic lateral meniscal abnormalities for UKA while excluding patients with more severe lateral-compartment pathology. Such selection may have resulted in confounding by indication and may partly account for the generally favorable outcomes observed in the LMI groups. Furthermore, adjustment for potentially important confounders, including age, body mass index, lower-limb alignment, severity of osteoarthritis, lateral cartilage status, implant design, and surgeon-related factors, was inconsistent across studies. Residual and unmeasured confounding therefore cannot be excluded. Retrospective assessment of preoperative imaging findings and postoperative clinical outcomes may also have introduced information or measurement bias. Consequently, the observed absence of an adverse association between lateral meniscal abnormalities and UKA outcomes should not be interpreted as definitive evidence of a causal absence of harm.

Second, substantial clinical heterogeneity existed in the definition and severity of lateral meniscal pathology. The included studies evaluated a spectrum of abnormalities, including meniscal tears, degenerative changes, intrameniscal signal abnormalities, meniscal extrusion, and histological calcification (3, 5–9, 11). Hurst et al. additionally used a broader definition of abnormal preoperative MRI findings rather than isolated lateral meniscal injury (10). The severity, tear morphology, stability, symptom status, and associated condition of the lateral articular cartilage were not consistently reported across studies. Consequently, the pooled analyses combine clinically heterogeneous forms of lateral meniscal pathology and cannot determine whether specific subtypes or more severe lesions carry different risks.

Third, direct evidence regarding symptom status was limited. Only two included studies explicitly evaluated patients with asymptomatic or clinically silent lateral meniscal abnormalities (5, 11), whereas symptom status was unclear or incompletely reported in the remaining cohorts. Therefore, the findings of this review should primarily be applied to appropriately selected patients with AMOA who lack clinically significant lateral joint-line pain, locking, or other mechanical symptoms and who have relatively preserved lateral-compartment cartilage. The findings should not be extrapolated to patients with symptomatic, unstable, traumatic, or root tears of the lateral meniscus or to those with advanced lateral-compartment cartilage loss.

Fourth, the sample sizes of the LMI groups were relatively small in several studies, and revision or implant failure events were uncommon, resulting in limited precision for rare outcomes. Follow-up duration also varied substantially, ranging from approximately 13.9 months to 10 years, with most studies providing only short- to mid-term observations. Shorter follow-up may adequately capture postoperative functional recovery but may underestimate late lateral-compartment osteoarthritis progression, aseptic loosening, polyethylene wear, and revision. Differences in implant design, particularly the predominance of mobile-bearing UKA in the quantitative analyses, further limit the generalizability of the pooled estimates. Finally, because fewer than 10 studies contributed to each pooled outcome, publication bias could not be reliably assessed using funnel plots or formal tests for asymmetry, and the possibility of publication bias cannot be excluded.

## 5 Conclusion

The available evidence suggests that asymptomatic or mild degenerative lateral meniscal abnormalities may not be associated with clinically important impairment in postoperative functional outcomes or knee range of motion after medial UKA in appropriately selected patients with AMOA. Therefore, the presence of an asymptomatic lateral meniscal abnormality on preoperative MRI alone should not necessarily be considered an absolute contraindication to medial UKA when the lateral articular cartilage is preserved. However, the certainty of evidence regarding postoperative lateral knee pain, revision or implant failure, and long-term radiographic progression of lateral-compartment osteoarthritis remains limited. Importantly, these conclusions should not be extrapolated to patients with symptomatic, unstable, traumatic, or root tears of the lateral meniscus or to those with advanced lateral-compartment cartilage loss. Further prospective studies using standardized definitions of lateral meniscal pathology and longer follow-up are warranted.
