# Supplementary material for: Impact of lateral meniscus injury on the efficacy of medial unicompartmental knee arthroplasty in patients with anteromedial osteoarthritis: a systematic review and meta-analysis
Source: Front Surg. 2026 Sep 22;13:1913901. doi: 10.3389/fsurg.2026.1913901 (PMC13640998; doi:10.3389/fsurg.2026.1913901)
Supplement: Supplementary file 1 [file Datasheet1.zip › Chart/search strategy.docx]

PubMed：

("lateral meniscus"[Title/Abstract] OR "lateral meniscal"[Title/Abstract] OR "lateral meniscus injury"[Title/Abstract] OR "lateral meniscal injury"[Title/Abstract] OR "lateral meniscus tear"[Title/Abstract] OR "lateral meniscal tear"[Title/Abstract] OR "lateral meniscus degeneration"[Title/Abstract] OR "lateral meniscal degeneration"[Title/Abstract] OR "lateral meniscus abnormality"[Title/Abstract] OR "lateral meniscal abnormality"[Title/Abstract] OR "lateral meniscus abnormalities"[Title/Abstract] OR "lateral meniscal abnormalities"[Title/Abstract] OR "lateral meniscus extrusion"[Title/Abstract] OR "lateral meniscal extrusion"[Title/Abstract] OR "lateral meniscus calcification"[Title/Abstract] OR "lateral meniscal calcification"[Title/Abstract] OR "lateral meniscal signal"[Title/Abstract]) AND ("unicompartmental knee arthroplasty"[Title/Abstract] OR "unicondylar knee arthroplasty"[Title/Abstract] OR "unicompartmental knee replacement"[Title/Abstract] OR "unicondylar knee replacement"[Title/Abstract] OR UKA[Title/Abstract] OR UKR[Title/Abstract] OR "partial knee replacement"[Title/Abstract]

Embase:

( 'lateral meniscus':ti,ab,kw OR 'lateral meniscal':ti,ab,kw OR 'lateral meniscus injury':ti,ab,kw OR 'lateral meniscal injury':ti,ab,kw OR 'lateral meniscus tear':ti,ab,kw OR 'lateral meniscal tear':ti,ab,kw OR 'lateral meniscus degeneration':ti,ab,kw OR 'lateral meniscal degeneration':ti,ab,kw OR 'lateral meniscus abnormality':ti,ab,kw OR 'lateral meniscal abnormality':ti,ab,kw OR 'lateral meniscus extrusion':ti,ab,kw OR 'lateral meniscal extrusion':ti,ab,kw OR 'lateral meniscus calcification':ti,ab,kw OR 'lateral meniscal calcification':ti,ab,kw OR 'lateral meniscal signal':ti,ab,kw ) AND ( 'unicompartmental knee arthroplasty':ti,ab,kw OR 'unicondylar knee arthroplasty':ti,ab,kw OR 'unicompartmental knee replacement':ti,ab,kw OR 'unicondylar knee replacement':ti,ab,kw OR uka:ti,ab,kw OR ukr:ti,ab,kw OR 'partial knee replacement':ti,ab,kw )

Web of Science Core Collection:

TS=( "lateral meniscus" OR "lateral meniscal" OR "lateral meniscus injury" OR "lateral meniscal injury" OR "lateral meniscus tear*" OR "lateral meniscal tear*" OR "lateral meniscus degeneration" OR "lateral meniscal degeneration" OR "lateral meniscus abnormalit*" OR "lateral meniscal abnormalit*" OR "lateral meniscus extrusion" OR "lateral meniscal extrusion" OR "lateral meniscus calcification" OR "lateral meniscal calcification" OR "lateral meniscal signal*" ) AND TS=( "unicompartmental knee arthroplast*" OR "unicondylar knee arthroplast*" OR "unicompartmental knee replacement*" OR "unicondylar knee replacement*" OR UKA OR UKR OR "partial knee replacement*" )

Cochrane Library:

#1 "lateral meniscus":ti,ab,kw #2 "lateral meniscal":ti,ab,kw #3 "lateral meniscus injur*":ti,ab,kw #4 "lateral meniscal injur*":ti,ab,kw #5 "lateral meniscus tear*":ti,ab,kw #6 "lateral meniscal tear*":ti,ab,kw #7 "lateral meniscus degenerat*":ti,ab,kw #8 "lateral meniscal degenerat*":ti,ab,kw #9 "lateral menisc* abnormal*":ti,ab,kw #10 "lateral menisc* extrusion":ti,ab,kw #11 "lateral menisc* calcif*":ti,ab,kw #12 #1 OR #2 OR #3 OR #4 OR #5 OR #6 OR #7 OR #8 OR #9 OR #10 OR #11 #13 "unicompartmental knee arthroplast*":ti,ab,kw #14 "unicondylar knee arthroplast*":ti,ab,kw #15 "unicompartmental knee replacement*":ti,ab,kw #16 "unicondylar knee replacement*":ti,ab,kw #17 UKA:ti,ab,kw #18 UKR:ti,ab,kw #19 "partial knee replacement*":ti,ab,kw #20 #13 OR #14 OR #15 OR #16 OR #17 OR #18 OR #19 #21 #12 AND #20

CNKI:

主题 = ( 外侧半月板 OR 外侧半月板损伤 OR 外侧半月板撕裂 OR 外侧半月板退变 OR 外侧半月板变性 OR 外侧半月板异常 OR 外侧半月板突出 OR 外侧半月板脱出 OR 外侧半月板挤出 OR 外侧半月板钙化 OR 外侧半月板异常信号 ) AND 主题 = ( 单髁置换 OR 单髁膝关节置换 OR 膝关节单髁置换 OR 单髁关节置换 OR 单间室膝关节置换 OR 膝关节单间室置换 OR UKA )

Wanfang Data:

( "外侧半月板" OR "外侧半月板损伤" OR "外侧半月板撕裂" OR "外侧半月板退变" OR "外侧半月板变性" OR "外侧半月板异常" OR "外侧半月板突出" OR "外侧半月板脱出" OR "外侧半月板挤出" OR "外侧半月板钙化" ) AND ( "单髁置换" OR "膝关节单髁置换" OR "单髁膝关节置换" OR "单间室膝关节置换" OR "UKA" )

VIP Database:

( 外侧半月板 OR 外侧半月板损伤 OR 外侧半月板撕裂 OR 外侧半月板退变 OR 外侧半月板变性 OR 外侧半月板异常 OR 外侧半月板突出 OR 外侧半月板挤出 ) AND ( 单髁置换 OR 膝关节单髁置换 OR 单间室膝关节置换 OR UKA )

Chinese Biomedical Literature Database (CBM)

( 外侧半月板 OR 外侧半月板损伤 OR 外侧半月板撕裂 OR 外侧半月板退变 OR 外侧半月板变性 OR 外侧半月板突出 OR 外侧半月板挤出 OR 外侧半月板钙化 ) AND ( 单髁置换 OR 膝关节单髁置换 OR 单间室膝关节置换 OR UKA )
