# Supplementary material for: Impact of lateral meniscus injury on the efficacy of medial unicompartmental knee arthroplasty in patients with anteromedial osteoarthritis: a systematic review and meta-analysis
Source: Front Surg. 2026 Sep 22;13:1913901. doi: 10.3389/fsurg.2026.1913901 (PMC13640998; doi:10.3389/fsurg.2026.1913901)
Supplement: Supplementary file 1 [file Datasheet1.zip › Chart/Table 1. Baseline characteristics of the included studies (n = 8).docx]

**Table 1. Baseline characteristics of the included studies (n = 8)**

| First Author (Year) | Country | Study Design | Sample Size (n) (Intact / LMI) | Mean Follow-up | UKA Implant Type | Outcome Measures |
| --- | --- | --- | --- | --- | --- | --- |
| Park S J(2021) | South Korea | Retrospective cohort | 125  (59/66) | 74.4 months | Fixed bearing | KSS, KOOS, lateral pain, squatting ability, survivorship, revision rate |
| Hernigou  P (2023) | France/China | Retrospective cohort | 130  (60/70) | 10 years | Fixed bearing | KSS, lateral radiographic progression (JSN), 10-year survivorship, revision rate |
| Zhu X (2023) | China | Retrospective cohort | 104  (59/45) | 39.2 months | Mobile bearing | HSS, ROM, lateral knee pain, squatting/extension ability, complications, revision rate |
| Hurst JM (2013) | USA | Retrospective cohort | 1000  (967/33) | 43.4 months (LMI) / 38.3 months (Intact) | Mobile bearing | KSS (pain, clinical, function), failure rate, revision rate |
| Fukai Y (2025) | Japan | Retrospective cohort | 117  (98/19) | > 3 years | Mobile bearing | OKS, KSS-F, ROM, lateral OA progression, revision rate |
| Tanaka T (2020) | Japan | Retrospective cohort | 102  (39/63) | 3 years | Mobile bearing | OKS, KSS-F, ROM, reoperation rate, revision rate |
| Chen H (2023) | China | Retrospective cohort | 101  (44/57) | ~2 years | Mobile bearing | AKSS-F, AKSS-O, OKS, ROM, HKA angle, complications, revision rate |
| Mao X  (2025) | China | Retrospective cohort | 30  (15/15) | 13.9 months | Mobile bearing | HSS, ROM, lateral knee pain, operative time, complications |

*Abbreviations:* **KSS**, Knee Society Score; **KOOS**, Knee injury and Osteoarthritis Outcome Score; **HSS**, Hospital for Special Surgery score; **OKS**, Oxford Knee Score; **KSS-F**, Knee Society Score-Function; **AKSS-F**, American Knee Society Score-Function; **AKSS-O**, American Knee Society Score-Objective; **ROM**, range of motion; **JSN**, joint space narrowing; **HKA**, hip-knee-ankle; **OA**, osteoarthritis.
