# Supplementary material for: Impact of lateral meniscus injury on the efficacy of medial unicompartmental knee arthroplasty in patients with anteromedial osteoarthritis: a systematic review and meta-analysis
Source: Front Surg. 2026 Sep 22;13:1913901. doi: 10.3389/fsurg.2026.1913901 (PMC13640998; doi:10.3389/fsurg.2026.1913901)
Supplement: Supplementary file 1 [file Datasheet1.zip › Chart/Table 2. Study-specific LMI phenotype and symptom status.docx]

**Table 2. Study-specific LMI phenotype and symptom status**

| Study | Original definition of LMI | Standardized phenotype | Severity information | Symptom status | Recommended use |
| --- | --- | --- | --- | --- | --- |
| Park 2021 | Stoller 1–3; degeneration/intrasubstance, horizontal, complex, radial, discoid lesions | Mixed degenerative signal + definite tear | Mixed; grade-specific outcome unavailable | Asymptomatic | Asymptomatic subgroup |
| Hernigou 2023 | Meniscal tear ± histological calcification | Tear / calcification | Phenotype-based, not ordinal | Unclear | Separate descriptive category |
| Zhu 2023 | Stoller grade 3 vs grade 0 | Definite structural tear | Grade 3: n=45 | Unclear | Definite-tear subgroup |
| Hurst 2013 | Abnormal preoperative MRI | Abnormal meniscus MRI | Mixed; grade-specific outcome unavailable | Unclear | Indirect evidence; separate descriptive interpretation |
| Fukai 2025 | Lateral meniscal extrusion | Extrusion | Grade 1: 18;  Grade 2: 1 | Unclear | Separate extrusion category |
| Tanaka 2020 | Stoller grades 1–3 | Degeneration / definite tear | G1=40, G2=15, G3=8 | Unclear | Severity analysis |
| Chen 2023 | Stoller grades 1–3 | Degeneration / definite tear | G1=38, G2=13, G3=6 | Unclear | Main severity-stratified study |
| Mao 2025 | Fischer II–III | MRI-defined structural LMI | II=2, III=13 | Asymptomatic | Asymptomatic subgroup |
