# Supplementary material for: Impact of lateral meniscus injury on the efficacy of medial unicompartmental knee arthroplasty in patients with anteromedial osteoarthritis: a systematic review and meta-analysis
Source: Front Surg. 2026 Sep 22;13:1913901. doi: 10.3389/fsurg.2026.1913901 (PMC13640998; doi:10.3389/fsurg.2026.1913901)
Supplement: Supplementary file 1 [file Datasheet1.zip › Chart/Table 3. GRADE assessment of the certainty of evidence for the main outcomes.docx]

**Table 3. GRADE assessment of the certainty of evidence for the main outcomes**

| Outcome | Studies / Participants | Effect estimate | Risk of bias | Inconsistency | Indirectness | Imprecision | Publication bias | Certainty |
| --- | --- | --- | --- | --- | --- | --- | --- | --- |
| Postoperative functional scores | 5 / 454 | SMD = −0.06 (95% CI −0.26 to 0.14) | Serious¹ | Not  serious² | Serious³ | Not  serious⁴ | Not formally assessed⁵ | Low ⊕⊕  ◯◯ |
| Postoperative knee ROM | 4 / 350 | MD = 0.17° (95% CI −1.58° to 1.92°) | Serious¹ | Not  serious² | Serious³ | Not  serious⁴ | Not formally assessed⁵ | Low ⊕⊕  ◯◯ |
| Postoperative lateral knee pain | 3 / 259 | RR = 1.10 (95% CI 0.32 to 3.81) | Serious¹ | Not  serious² | Serious³ | Serious⁶ | Not formally assessed⁵ | Very low ⊕◯  ◯◯ |
| Revision or implant failure | 7 / 1,679 | No reliable pooled estimate; descriptive synthesis | Serious⁷ | Not  assessable⁸ | Serious⁹ | Very serious¹⁰ | Not formally assessed⁵ | Very low ⊕◯  ◯◯ |
| Radiographic progression of lateral-compartment OA | 3 / 349 | No reliable pooled estimate; descriptive synthesis | Serious¹ | Not  assessable⁸ | Serious³ | Very serious¹⁰ | Not formally assessed⁵ | Very low ⊕◯  ◯◯ |

**¹ Risk of bias:** Downgraded by one level because all contributing studies were retrospective cohort studies, with potential selection bias, residual confounding, and inconsistent adjustment for relevant prognostic variables.

**² Inconsistency:** Not downgraded because statistical heterogeneity was negligible (I² = 0%) and no important inconsistency in the direction of effects was identified.

**³ Indirectness:** Downgraded by one level because the definition of lateral meniscal abnormality varied across studies, including meniscal tears, degenerative changes, intrameniscal signal abnormalities, meniscal extrusion, and histological calcification.

**⁴ Imprecision:** Not downgraded because the confidence intervals were relatively narrow and did not encompass large clinically important effects.

**⁵ Publication bias:** Formal assessment using funnel plots or statistical tests for funnel-plot asymmetry was not performed because fewer than 10 studies contributed to each quantitative outcome. Publication bias therefore could not be reliably assessed, and no additional downgrading was applied solely on this basis.

**⁶ Imprecision:** Downgraded by one level because the confidence interval was wide and encompassed both a potentially clinically important reduction and increase in risk.

**⁷ Risk of bias for revision or implant failure:** Downgraded by one level because the evidence was derived entirely from retrospective cohort studies, with potential selection bias and residual confounding, and rare-event estimates were particularly vulnerable to instability.

**⁸ Inconsistency:** Could not be reliably assessed because the outcome was synthesized descriptively rather than quantitatively and the available event data were sparse.

**⁹ Indirectness for revision or implant failure:** Downgraded by one level because Hurst et al. evaluated a broader category of abnormal preoperative MRI findings rather than isolated lateral meniscal injury.

**¹⁰ Very serious imprecision:** Downgraded by two levels because of the very small number of outcome events and insufficient information to exclude clinically important increases or decreases in risk.
